# Population Genetics of *Trypanosoma evansi* from Camel in the Sudan

**DOI:** 10.1371/journal.pntd.0001196

**Published:** 2011-06-07

**Authors:** Bashir Salim, Thierry de Meeûs, Mohammed A. Bakheit, Joseph Kamau, Ichiro Nakamura, Chihiro Sugimoto

**Affiliations:** 1 Department of Collaboration and Education, Research Center for Zoonosis Control, Hokkaido University, Sapporo, Japan; 2 Department of Parasitology, Faculty of Veterinary Medicine, University of Khartoum, Khartoum-North, Sudan; 3 UMR IRD/CIRAD 177 INTERTRYP, CIRDES, Bobo-Dioulasso, Burkina-Faso; 4 CNRS, Délégation Languedoc-Roussillon, Montpellier, France; 5 Veterinary Infection-Biology and -Immunology, Research Center Borstel, Borstel, Germany; IRD/CIRDES, Burkina Faso

## Abstract

Genetic variation of microsatellite loci is a widely used method for the analysis of population genetic structure of microorganisms. We have investigated genetic variation at 15 microsatellite loci of *T. evansi* isolated from camels in Sudan and Kenya to evaluate the genetic information partitioned within and between individuals and between sites. We detected a strong signal of isolation by distance across the area sampled. The results also indicate that either, and as expected, *T. evansi* is purely clonal and structured in small units at very local scales and that there are numerous allelic dropouts in the data, or that this species often sexually recombines without the need of the “normal” definitive host, the tsetse fly or as the recurrent immigration from sexually recombined *T. brucei brucei*. Though the first hypothesis is the most likely, discriminating between these two incompatible hypotheses will require further studies at much localized scales.

## Author Summary

Trypanosomiasis due to *Trypanosoma evansi* is a widely distributed disease of livestock, affecting especially camelids and equines and is transmitted by biting flies. The disease is of great concern to many developing countries such as Sudan, where its large camel population estimated at over 4.6 million heads is at risk. It is generally believed that *T. evansi* has evolved when camels infected with *T. brucei* moved to tsetse-free areas, but only a few studies have been carried out to elucidate the genetic make-up of *T. evansi*. Therefore, in the current study, 15 microsatellite markers from non-coding loci on 38 isolates of *T. evansi* originating from different locations in Sudan were analyzed. Three reference strains from Sudan and Kenya were additionally analyzed and compared to the recent isolates. The results of this study revealed a highly significant isolation by distance pattern with rather small neighborhood sizes. It also suggested that *T. evansi* is either purely clonal with numerous problems of allelic dropouts or that it often sexually recombines without the need of the definitive host, the tsetse fly, or as the result of recurrent immigration from sexually recombined *T. brucei brucei* mutants.

## Introduction

*Trypanosoma evansi* is the most widely distributed of the pathogenic animal trypanosomes, affecting domesticated livestock in Asia, Africa and Central, South America, Canaries Island and recently in Europe (Spain and France) [1–2–3–4]). Until recently, countries were obligated to declare to the OIE surra outbreaks only in equine species, while infection in other animal species was excluded. Recently, this limitation was modified at the OIE Executive Committee meeting held in May 2008: surra is now considered an “OIE listed disease – multi-species” – to be reported to the OIE in the same way as the previously listed trypanosomes (Dourine, surra in horses, tsetse transmitted trypanosomoses) (see: OIE Manual of Diagnostics for terrestrial animals, edit. 17 July 2008, online). Recently human infections have been reported in India making it a potential human pathogen [5].

Camel trypanosomiasis caused by *T. evansi* is of great concern to countries like Sudan, which possesses the second largest camel population in the world, estimated at nearly 4,623,000 heads (Annual Report of Federal Ministry of Animal Resources and Fisheries, Sudan, 2010). The existence of carrier animals in the vicinity of susceptible camels makes transmission by biting flies possible. The old paradigm according to which *T. evansi* evolved, via *T. equiperdum*, when camels infected with *T. brucei* moved to tsetse free areas [6] is now challenged by the consensus of opinion that both evolved from *T. brucei brucei* [7]. It was even more recently suggested that *T. evansi* should be considered as a subspecies of *T. brucei* complex [8–9–10].

Microsatellite-length polymorphisms using PCR (microsatellite loci) have been recently and widely used for molecular typing of genetically distinct parasite populations such as *Plasmodium* spp. [11]–[12], *Theileria parva* [13], *Cryptosporidium parvum* [14], *Toxoplasma gondii* [15]–[16], *Leishmania* spp. [17–18–19], *Trypanosoma cruzi* [20]–[21] and *Trypanosoma brucei* groups [22–23–24]. Microsatellite markers have been shown to be polymorphic enough to highlight the existence of genetic diversity within the very homogeneous *T. evansi* [25]–[26].

In Sudan, a few molecular studies have been carried out on *T. evansi* using isoenzyme characterizations [8] or on drug resistance of *T. evansi* [27]–[28]. Parasite prevalence and infection pattern were also performed with varying estimates of prevalence 5.4% using parasitological examination [29], 31.3% with ELISA [29]. The overall prevalence estimated by Salim et al., using molecular epidemiological tools, ranged between 33.9 to 42.1% [30]. However basic genetic analyses of the parasite populations in the country using multilocus neutral markers have not been reported so far.

In this study we selected 15 microsatellite markers from non-coding loci (a priori not subjected to selective forces) on 38 isolates from different sites from Sudan and three reference strains from Sudan and Kenya. This is the first report on microsatellite markers from non-coding loci in population analysis of *T. evansi* in east Africa.

## Materials and Methods

### Trypanosomes samples

**Figure 1:**
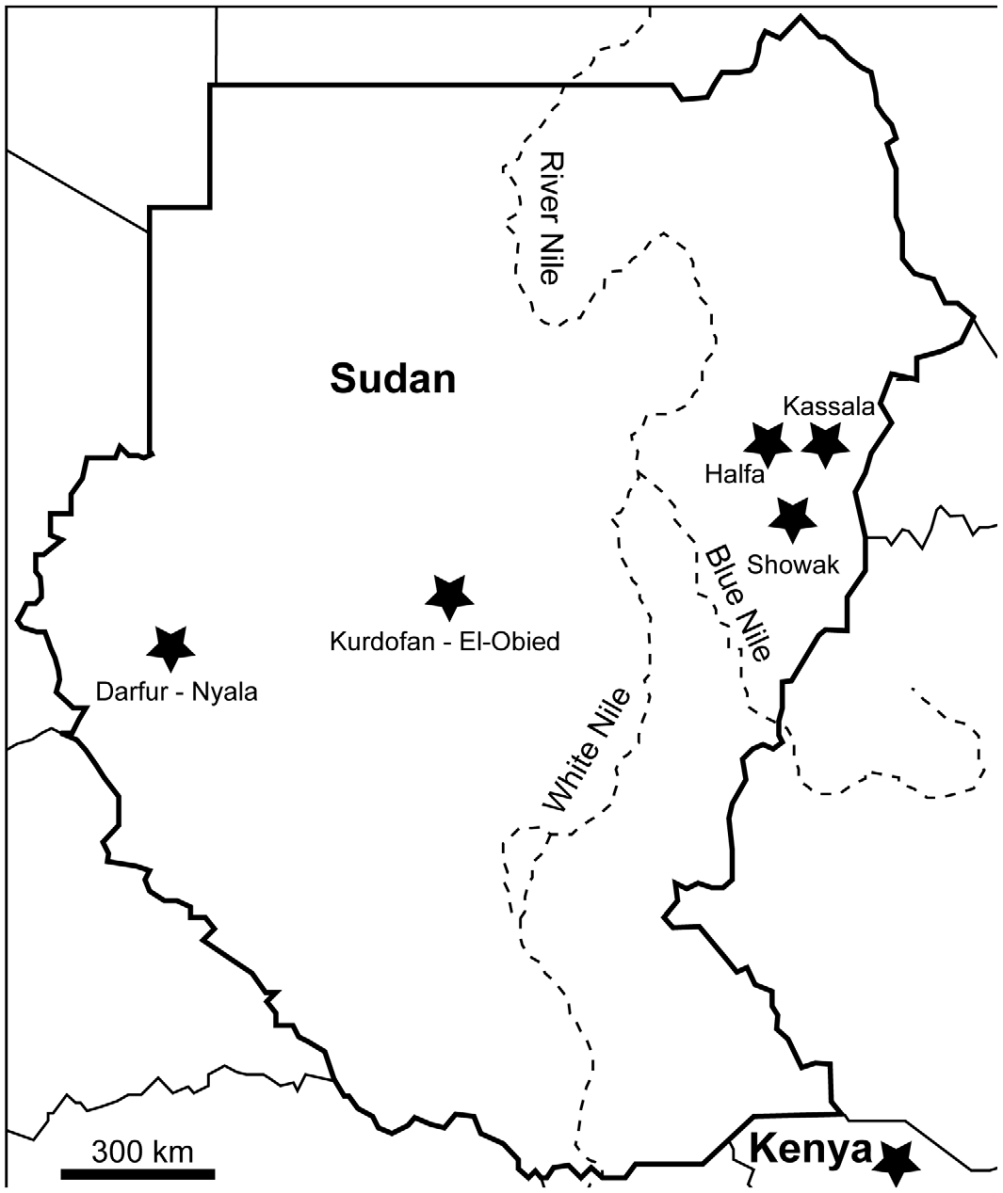
Localization of sampling areas within Sudan and Kenya.

A total of 685 samples were collected during a survey conducted in March 2008 and during the period between September and November 2009 from seven geographically distinct zones in Sudan (Figure 1). These regions were grouped as “West Nile and East Nile regions” their names and location coordinates are shown in Table 1. Samples were collected from different camel herds mostly nomads that perform transhumance northwards migration in the wet season and southwards in the dry seasons. Sixty two *T. evansi* positive samples were included in this study in addition to three reference samples collected previously from Sudan and Kenya (Table 1). To maintain anonymity of subject and owners' confidentiality and to adhere to the International Ethical Guidelines for Biomedical Research involving animal subjects, no owner names were recorded within the database or as part of the data collection process. The owners of the sampled camels provided consent to have their animals included in the study. Research on samples from animals was conducted adhering to guidelines of the Institutional Animal Care and Use Committee of the Graduate School of Veterinary Medicine, Hokkaido University. The study protocol has also been approved by the Faculty of Veterinary Medicine, University of Khartoum, according to their guidelines for sampling domestic animals in Sudan.

**Table 1:** Subsamples, zone (East or West Nile), site and year of sampling, GPS coordinates and number of genotyped isolates (N) of *T. evansi.*

Zone	Site	Year	Latitude	Longitude	Subsample	N
East	SUDAN	1976*	Unknown	Unknown		1
East	KENYA	1978*	Unknown	Unknown		1
East	KENYA	1980*	Unknown	Unknown		1
East	Kassala	2008	15°30′N	36°00′E	2	3
West	Kurdofan	2008	13°08′N	30°10′E	3	3
East	Showak	2009	14°02′N	35°28′E	1	7
East	Halfa	2009	15°19′N	35°36′E	4	6
East	Kassala	2009	15°30′N	36°00′E	5	4
West	Darfur	2009	12°02′N	24°58′E	6	10
West	Kurdofan	2009	13°08′N	30°10′E	7	5

*These three reference strains were obtained from International livestock research institute (ILRI).

### DNA extraction

Fourteen trypanosome isolates were purified from blood cells using DE-52 columns (Whatman, UK) and trypanosome DNA was extracted using Qiamp DNA mini kit (Qiagen, Australia) following manufacturer's instructions then stored at −20°C. Forty eight more samples were collected in FTA cards (Whatman FTA Classic Cards, Whatman,UK), and the DNA was eluted from blood spotted onto the FTA cards using a modified methanol fixation method as described by [31]. Isolated DNA was stored at −20°C until used.

### PCR

All samples were first subjected to a PCR test, which amplified the ITS1 region of rRNA gene of all African trypanosomes according to [32]. To exclude other *T. brucei* subspecies, samples were further analyzed with a PCR test specific for *T. evansi*, using a primer set that amplified 151bp of the *T. evansi* RoTat gene fragment [33]. The primer set used was TeRoTat920F 5′-CTG AAG AGG TTG GAA ATG GAG AAG-3′ and TeRoTat1070R, 5′-GTT TCG GTG GTT CTG TTG TTG TTA-3′ and the reaction conditions were set in a final volume of 20 µl, which contained 50 ng/µl templates DNA, 10 mM primer “forward and reverse” added to AmpliTaq Gold DNA Master Mix. The cycling conditions consisted of initial step at 94°C for 5 min, followed by 35 cycles of 94°C for 40 s, 58°C for 40 s, 72°C for 1 min, and final extension at 72°C for 5 min. All 62 samples were verified as *T. evansi*.

Out of the 62 samples examined, 41 were successfully analyzed for 15 microsatellite markers. These markers were selected from the *T. brucei* genome project release 4. The markers used here were originally designed *in silico* and computed by [34]. The 15 markers are distributed across all 11 chromosomes of the *T. brucei* and no two loci are closely physically linked. Markers 10/1, 10/5 and 10/19 on chromosome 10, are separated by distance of 84 Kb and 2.2 Gb respectively, while 11/13 and 11/29, on chromosome 11, are separated by distance of 48 Kb. Recombination rate is in average 15.6 kb/cM in *T. brucei* according to reference [34], making that a distance of 48 kb is unlikely to create a strong physical linkage. Markers used and different loci are shown in Table 2. The loci were amplified using the following PCR conditions: initial denaturation step at 94°C for 10 min, followed by 35 cycles of 94°C for 30 s, annealing for 30 s, 72°C for 1 min, and final extension at 72°C for 5 min. The annealing temperatures used for different microsatellite amplification are indicated in Table 2. PCR products were electrophoresed in 2% agarose in TAE buffer and stained using GelRed dye (Biotium, USA) before being visualized under UV light.

**Table 2:** Primer sequences used for amplification of microsatellite loci.

Locus	Chromosome	Primer sequence	Repeat	T_A_ (°C)	References
TB1/8	1	F: AGGTTTAGTGCATGTCGGAR: CCTGTTGTACGGAGGTCA	(CA)n	53	Macleod et al. 2005
TB2/19	2	F: CTGGTGCGTGTAACTGTGR: GAAGTGAGGACATGCACG	(AT)n	53	” ”
TB5/2	5	F: CAACCGAAAGTAAGGGGAACR: TCTCGCCTTCTTTGCCC	(AT)n	60	” ”
TB6/7	6	F: AAGCTGACAGGTGGTTGAR: GAACATGCGTGCGTGTG	(AT)n	53	” ”
TB8/11	8	FP: TGTAGCAGTGGTACGCACRP: CACCCAACGCATGTAAGC	(AT)n	53	” ”
TB10/5	10	F: AAAGGCGATATGTTATTATTGAR: ATTGGGTATACTGTCCCTCA	(TA)	60	” ”
TB11/13	11	F: CAAGAACTCTGCATTGAGCR: ATCTGTTGGCGATGGTGA	(AT)n	53	” ”
TB10/19	10	F: CTGTTCGTTCTGAATTGTGTGCGR: GTGCACTTCCTTCTCTCATCCTTTTC	(CA)n	56	” ”
TB10/1	10	F: GCTCTACGCACCCACACAATCCGTR: CTCACTTGAGTAACCTCTCATTGC	(CAA)n	56	” ”
TB11/29	11	F: AATGAGTGATACTATGAAAGTGTR: CACCATCACTGCTCTTATCA	?	56	” ”
TB11/1	11	F: AGTATGTGTGGACAGTTAAGAR: ACCTTTGAGTCTTTCCTGTT	(TA)n	56	” ”
TB3/3	3	F: CATTCGAAGTAAATGCGCGTATAACR: GGTTGGAGCTTTCGACACAAGCG	(AT)n	56	” ”
TB4/2	4	F: GCCGCTTGATCATTAGGTAACCACR: CCGCCTCACTTTAAGGATGGTGCC	(CA)n	56	” ”
TB7/12	7	F: CATGGCGTACGTTGCTTCGGTTTCR: GGTCGGTGTTGGCAGTGTGCATAG	(GT)n	56	” ”
TB8/1	8	F: CCAAATATGCGATTAGTTTCCR: TGTTTATGTGGAAGGAAATGAA	(TA)n	56	” ”

All forward primers were labeled with fluorescent dye at the 5′ termini. Following multiplex PCRs with the forward fluorescent labeled primer and the unlabeled reverse primer (reverse primers are modified to add A to PCR-products, check the ABI website), as described above, 1 to 5 µl of each product was mixed with 9 µl formamide and 0.5 to 1 µl of GeneScan 600 LIZ size standard (Applied Biosystems, USA). The samples were denatured at 95 °C for 3 min and cooled prior to electrophoresis on an ABI PRISM 3130 Genetic Analyzer under denaturing conditions on a 50 cm capillary column with performance-optimized pop 7 polymer (Applied Biosystems). The instrument was previously calibrated with DS-33 matrix standards (Applied Biosystems). The electrophoresis data were analyzed with GeneMapper software v4.0 (Applied Biosystems).

### Genetic data analysis

For most of analysis, we used Create v1.1 [35] from a text file general spreadsheet to convert it into the appropriate format. Because genetic differentiation occurs at a spatial and at a temporal scale in Trypanosomatidae [23–19], we distinguished each of the seven year×site combinations as separated subsamples (see Table 1). Reference strains from Sudan 1976, Kenya 1978 and Kenya 1980 were only considered for the Neighbor-Joining Tree (NJTree) analyses (see below). Number of strains, GPS coordinates and year of sampling can be seen in Table 1.

Linkage disequilibrium was tested with *G*-based randomization test implemented in Fstat 2.9.3.2. [36], updated from [37] per pair of loci and overall subsamples as recommended [38]. There are as many tests as possible pairs of loci (here 15×14/2 = 105). We thus expect 0.05×105∼5 significant tests under the null hypothesis of no linkage with α = 0.05. We thus tested if there was significantly more than 5% significant tests in the 105 tests series with a unilateral exact binomial test under R [39] to test for the existence of a global signal across the whole test series (hence the genome) and used the sequential Bonferroni procedure [40]–[41] by multiplying the smallest *P*-value by the number of remaining tests to identify which tests are significant (e.g. [42]).

Wright's *F*-statistics [43], the parameters most widely used to describe population structure [44], were initially defined for a three-level hierarchical population structure (individuals, sub-populations and total). In such a structure, three fixation indices or *F*-statistics can be defined. *F*_IS_ is a measure of the inbreeding of individuals (hence I) resulting from non-random union of gametes within each sub-population (hence S). *F*_ST_ is a measure of the relatedness between individuals resulting from non-random distribution of individuals among sub-populations, relative to the total population; *F*_ST_ quantifies the differentiation between sub-populations in the total population (hence S and T). These *F*-statistics are classically estimated by Weir and Cockerham's unbiased estimators *f* (for *F*_IS_) and *θ* (for *F*_ST_) [45]. These statistics were estimated with Fstat 2.9.3.2. *F*_IS_ is particularly convenient to measure departure from panmixia and in particular clonal reproduction that is expected to generate strongly negative values at all loci when predominant [46–47–48]. *F*_ST_ is a convenient measure of differentiation between the different sub samples of a data set. Its estimator is expected around 0 under the null hypothesis of random distribution of genotypes across sub samples and positive values, up to 1, in case of genetic differences. The significant deviation from 0 was tested through randomization of alleles between individuals within sub samples for *F*_IS_ and the statistic used was *f*; the estimator of *F*_IS_. In all cases, randomisation number was set to 10000. These tests are unilateral. For *F*_IS_, because in partially clonal organisms it can display positive and negative values, a bilateral test, testing if *F*_IS_ values are not significantly above or below 0, must be implemented. This is simply implemented by using the *P*-values obtained with Fstat when testing for *F*_IS_>0 and *F*_IS_<0 and computing *P*_bilateral_ = *P*_min_+(1-*P*_max_) and where *P*_min_ and *P*_max_ are the two *P*-values obtained and *P*_min_ is the smallest one. When individual tests were needed, to identify which loci are in departure from the *F*_IS_ expected under random union of gametes (panmixia), we also used the sequential Bonferroni procedure. 95% confidence intervals of *F*-statistics were computed by bootstrap over loci for the mean, and by jackknife over populations for individual loci undertaken with Fstat 2.9.3.2 [49].

A convenient way to represent the genetic composition of clonal organisms is to draw a dendrogram based on genetic distances. We computed Cavalli-Sforza and Edwards [50] chord distance matrices between all individual strains with MSA [51] (and drew a Neighbour-Joining Tree (NJTree) with Mega 3 [52] as recommended [53].

Isolation by distance was assessed following Rousset's method (Rousset, 1997)[54] using *F*_ST_/(1-*F*_ST_) as the genetic distance to regress against geographic distance *D_G_*. For a two dimensional model of population structure, neighborhood size is related to the slope *b* of the regression between geographic distances natural logarithm Ln(*D_G_*) (computed out of the georeferenced coordinates of each isolate) with the equation *F*_ST_/(1-*F*_ST_) = *b*×Ln(*D_G_*)+Constant, with *b* = 1/4*πD_e_σ* ^2^, and where *D_e_σ* ^2^ is the product of the effective population density (i.e. ∼density of reproducing adults per square meter) by the dispersal surface that separates them from their parents [54]. In a two dimensional framework the product *N_e_m* of the effective population size times the migration rate, which corresponds to the number of migrants arriving in one neighbourhood from the other neighboring sites, is equal to *N_e_m* = 2*Dσ* ^2^ = 1/(2*πb*) [54]. The significance of the regression was tested by a Mantel test [55] with 1000000 randomizations (Markov chain method) and 95% confidence intervals (CI) by bootstrap over loci. Isolation by distance procedures and testing were all implemented using Genepop version 4 [56]. For this test, only subsamples from year 2009 were considered in order to avoid temporal Wahlund effects [23–19].

## Results and Discussion

### Linkage disequilibrium

**Figure 2:**
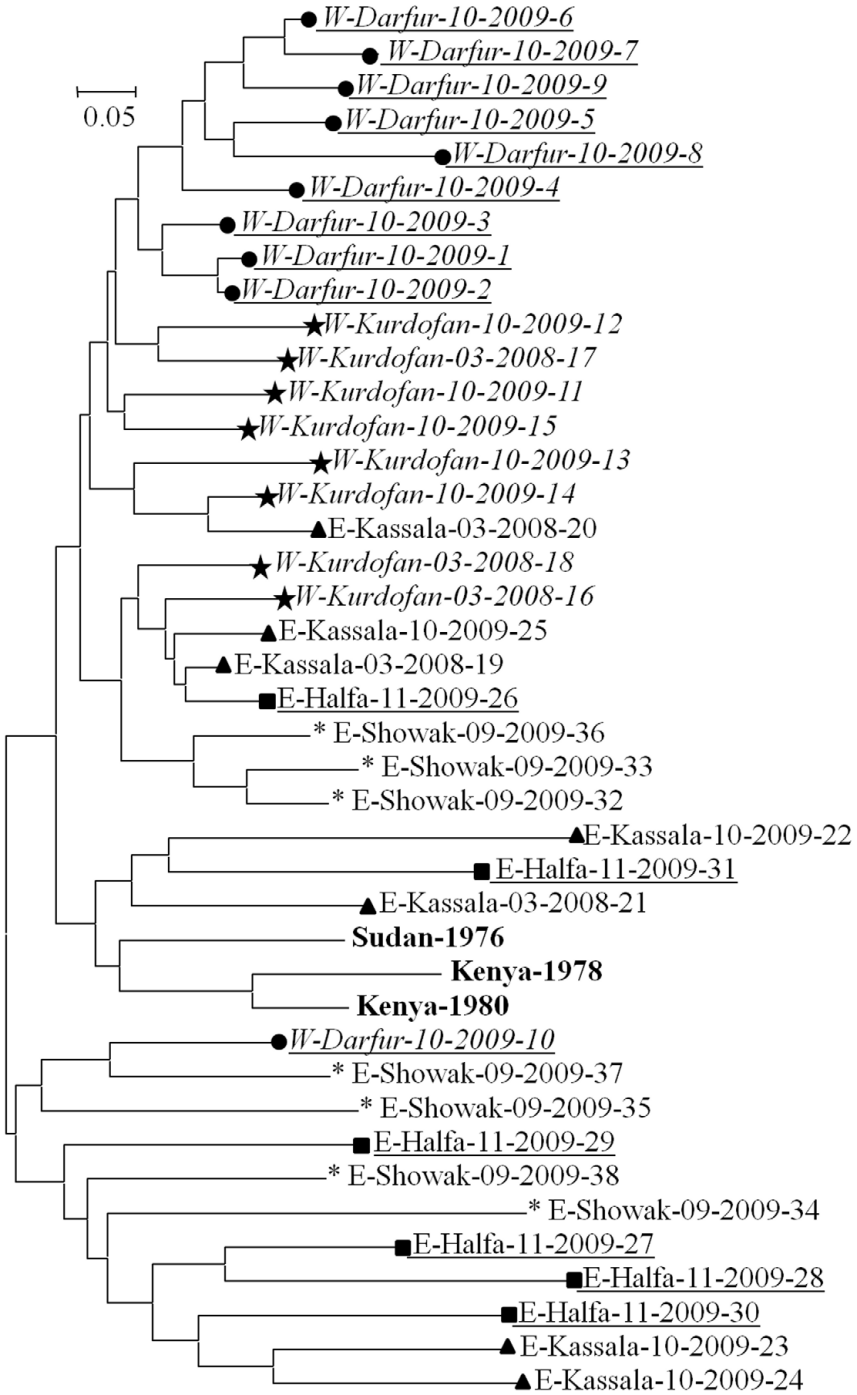
Unrooted NJTree between the different strains of *T. evansi*. The tree is based on Cavalli-Sforza and Edward's chord distance matrix computed over all the 15 loci. Name of the strains: first letter side as regard to Nile, site name, month, year of sampling and number of the strain. Reference strains are in bold. The different sites are represented with different symbols.

Among the 105 possible pairs of loci, 13 were in significant linkage at the 5% level of significance. This if far above the 5 expected under the null hypothesis (*P*-value = 0.0023). None of these pairs stays significant after Bonferroni correction, but this is probably due to the low power of individual tests and the extreme severity of correction (first lowest *P*-value must be under or equal to 0.00048 to stay significant here) [see [49]). We can nevertheless note that some loci tended to appear in significant linked pairs more often than expected if linkage was due to the reproductive system or population structure (leading to balanced genome wide signatures). For instance, loci TB10/5 and TB6/7 were both found five times in significant linkage. Here with 105 possible locus pairs, 14 possible pairs per locus (*P*_1_ = 14/105) and 13 significant tests (*P*_2_ = 13/105), we expect each locus to be in significant linkage with probability *P*_3_ = *P*_1_×*P*_2_ = 0.017. An exact binomial test with five successes among 105 attempts, mean probability = 0.017 and alternative hypothesis “greater” gives a significant result for loci TB10/5 and TB6/7 (*P*-value = 0.031). Physical linkage is not a really satisfactory explanation as other loci involved in linkage with these two loci should also be linked which is not often the case here, but this cannot be excluded. Moreover, and unless chromosomal changes occurred between *T. brucei* and *T. evansi*, all markers are distributed across all 11 chromosomes of the *T. brucei* and no two loci are closely physically linked (see Materials and Methods). It is also noteworthy that no multilocus repeated genotype (MLG) could be found across the whole data set, which is unexpected if *T. evansi* is clonal. This unusual genetic structure can be illustrated by the NJTree in Figure 2. Huge effective population size could maintain a great diversity of MLGs but would have prevented isolation by distance pattern to emerge (see below). Mutation rate would need to be extreme also to totally hinder repeated MLGs clonal signatures over all subsamples.

### NJTree analysis

In the dendrogram of Figure 2, West Nile strains seem reasonably together, while East Nile strains appear more heterogeneous. Despite one outlier, Darfur strains are well gathered, and then Kurdofan. Most Showak strains are gathered in Eastern Nile cluster, but again with strong heterogeneity, some being clustered in the “wrong” side of Nile. Kassala and Halfa strains can be found almost everywhere. Globally, considering that Cavalli-Sforza and Edwards chord distance is bounded to 1, the differentiation between strains of the present data is very strong and the distance between some isolates could have been measured between different taxa.

### Isolation by distance

**Figure 3:**
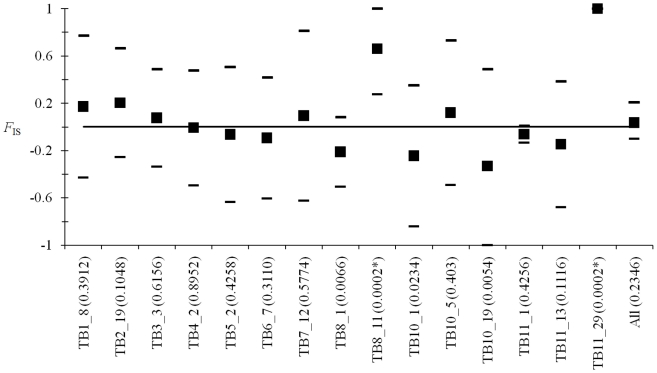
Isolation by distance between *T. evansi* from Sudan 2009. Equations are of the form *F*_ST_/(1-*F*_ST_)∼*b*LN(*D_G_*)-0.233, where *F*_ST_ is Wright's fixation index estimated with Weir and Cockerham's method and *b* is the slope of the regression and is equal to 0.067 for the main regression and *b*1 = 0.032 and *b*2 = 0.137 for the 95% confidence interval slopes. The relationship was highly significant (*P*-value = 0.008, Mantel test, 10^6^ randomizations).

There is a significant isolation by distance (*P*-value = 0.008) with a slope *b* = 0.0668 with 95% CI = [0.03, 0.14] (Figure 3). This would correspond to a strongly viscous population (at the scale investigated) with neighborhood size *Nb* = 15 individuals and small number of immigrants *N_e_m* = 2.4 individuals per generation. Obviously, even if camels are known to migrate much over the entire zone and even from other countries of the region [29], infected camels either do not migrate and/or end with poor local transmission to autochthonous hosts when immigrating in new sites and/or insects vector transmit the parasites mainly within herds and not between herds.

### Heterozygosity analysis

**Figure 4:**
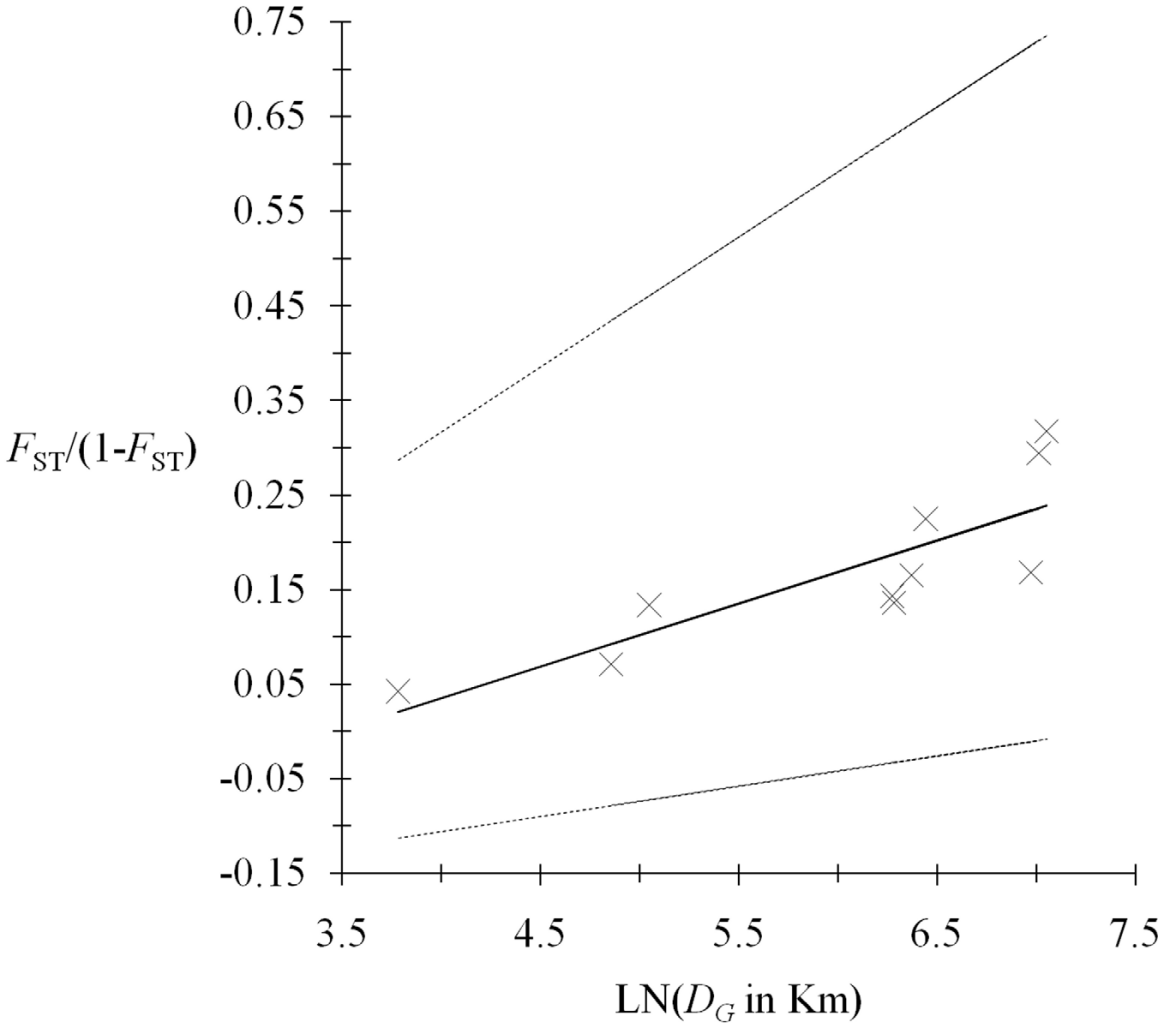
*F*_IS_ analysis for each locus and overall (All) of *T. evansi*. Individual bilateral *P*-values are indicated within brackets. Those significant after sequential Bonferroni correction are indicated with a *.Mean *F*_IS_ were computed over all subsamples (as defined in the text) and 95% confidence intervals computed using jackknife over subsamples (see De Meeûs et al., 2007 [49]), except for the global mean over loci (bootstrap over loci).

*F*_IS_ analysis results are presented in Figure 4. Over all loci, there is an apparent agreement with panmixia (*F*_IS_∼0, *P*-value = 0.23). This, with an apparent absence of MLG, might be the signature of frequent sex in *T. evansi*. Nevertheless, some loci display strongly and significant heterozygote excesses or deficits, some of which stay significant at the sequential Bonferroni level. This is not expected under panmixia. From here, several questions arise. First, *T. evansi* has lost the ability to transform into specific stages that colonize tsetse midgut and salivary glands where sexual recombination occurs and the existence of sexual recombination is thus highly unlikely in this species [57]–[58]. Moreover, other population genetic studies [22]–[26] reveal that levels of heterozygosity of some markers can be very high, that missing allele may be frequent and MLGs numerous. In fact, missing DNA can be found in diploid organisms that are not constrained to frequent meiosis, like it is the case in the yeast *Candida albicans* [59] or in *Leishmania* [60]. These DNA losses, if frequent enough, may lead to frequent haploid-like sequences that are in fact heterozygous “DNA/Missing DNA” and, combined with Wahlund effects, could have led to the odd results obtained during linkage disequilibrium and *F*_IS_ analyses. Allelic dropout and gene conversion can have similar consequences and do occur in kinetoplastid parasites [61]–[62]. The fact that primers were designed from another species, *T. brucei*, could indeed have lead to discrepancies in microsatellite loci flanking sequences and hence to allelic dropouts and/or null alleles.

We undertook a quick and incomplete simulation study with Easypop v2.01 [63] to check if a Wahlund effect combined with allelic dropout could lead to our observations. It appeared that this indeed can be achieved with certain parameter sets (see Supplementary Material S1) and thus that our interpretation regarding a combined effect of some Wahlund effects and allelic dropouts is rather reasonable. Another source of Wahlund effect would come from the frequent migration of infected camels from different areas. Camels are indeed known to move a lot in this part of Africa [29]. Nevertheless, this hypothesis is extremely hard to reconcile with the clear isolation by distance found that would surely be destroyed by such massive migration of parasite strains. This hypothesis is also self contradictory as even such Wahlund effect would quickly vanish due to the homogenization of parasite populations that such migration pattern would produce, unless one admits these migrating strains are unable to survive locally for unknown reasons. Given the formidable spread this parasite has experienced across the world [4], such an explanation appears poorly convincing.

Another interpretation would come from [10] and would mean *T. evansi* stocks are continuously filled with recombining *T. brucei brucei* that would recurrently loose their maxicircle kDNA. This hypothesis requires that this phenomenon be enough frequent so that “brucei” and “evansi” stocks display exactly the same isolation by distance pattern. If this was true “evansi” stocks would never happen to cluster with any kind of marker. This is apparently not obviously the case as *T. evansi* strains can cluster together pretty well [64–65–66–58]. Moreover, we need a rather strong Wahlund effect to explain the shape of the NJTree under such a recurrent recombination events hypothesis. Though we cannot reject totally this hypothesis, it is not the most parsimonious and we will not consider it further.

### Conclusion

If we do not assume full clonality for *T. evansi* our genetic data are difficult to interpret and in contradiction with what is expected from this strictly mechanically transmitted trypanosome [58]. Moreover, the shape of the NJTree obtained is compatible with frequent allelic dropouts, Wahlund effects or both. Sampling at much smaller scales and redesigning primers would offer the opportunity to test these hypotheses. If true, this would mean much smaller (more negative) *F*_IS_ to be considered. In the opposite case, the possibility of recombination for *T. evansi* freed from the tsetse obligate stages or recurrently coming from mutant *T. brucei brucei* could be considered though we do not consider it as a likely explanation. This remains to be investigated further including *T. brucei brucei* strains in sites where both species can be found, which is hardly the case here as most our samples are North of the tsetse belt. This is an important issue as these different hypotheses (full clonality and strong viscosity versus sexual reproduction) have not the same consequences in term of spread of parasite resistance to trypanocidal drugs. Nevertheless, isolation by distance was evidenced with a relatively small number of migrants between neighboring sites at each generation. Given the strong structuring power of stepping stones, this means quite a strong viscosity as regards to parasite propagation of *T. evansi* across camel herds in Sudan.

## Supporting Information

Supplementary Material S1 Description of sampling designs, Wahlund protocols, dropout protocols and results of simulations for testing how Wahlund effects and/or allelic dropouts can help interpreting *Trypanosoma evansi* data in Sudan.
(DOC)
