# Correction: Population Genetics of *Trypanosoma evansi* from Camel in the Sudan

**DOI:** 10.1371/journal.pntd.0003417

**Published:** 2014-12-01

**Authors:** The PLOS Neglected Tropical Diseases Staff

The figure legends for Figure 3 and Figure 4 are currently switched. Please find the corrected figure legends here.

**Figure 3:**
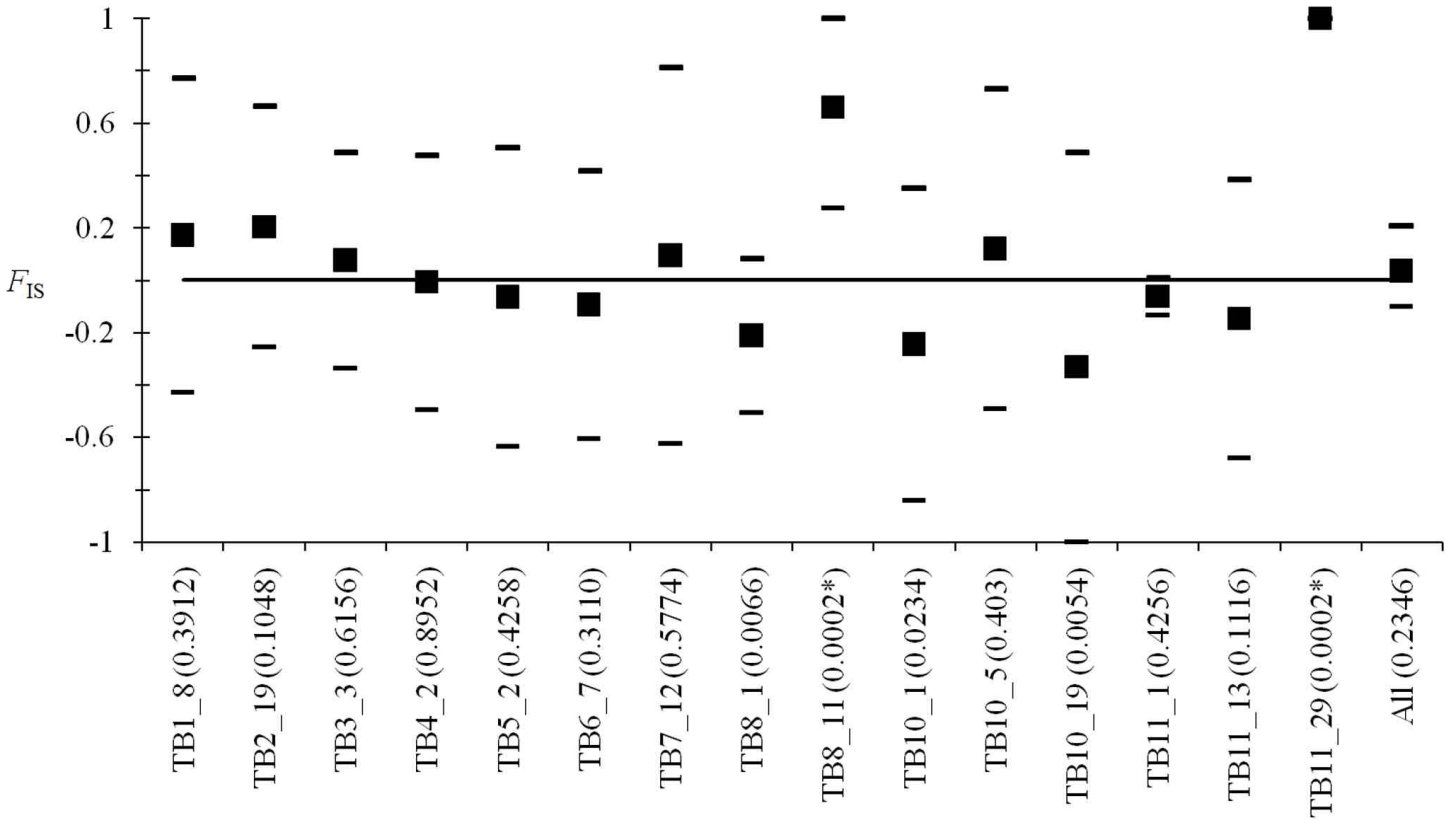
*F_IS_* analysis for each locus and overall (All) of *T. evansi*. Individual bilateral *P*-values are indicated within brackets. Those significant after sequential Bonferroni correction are indicated with a *.Mean *F*_IS_ were computed over all subsamples (as defined in the text) and 95% confidence intervals computed using jackknife over subsamples (see De Meeûs et al., 2007 [49]), except for the global mean over loci (bootstrap over loci).

**Figure 4:**
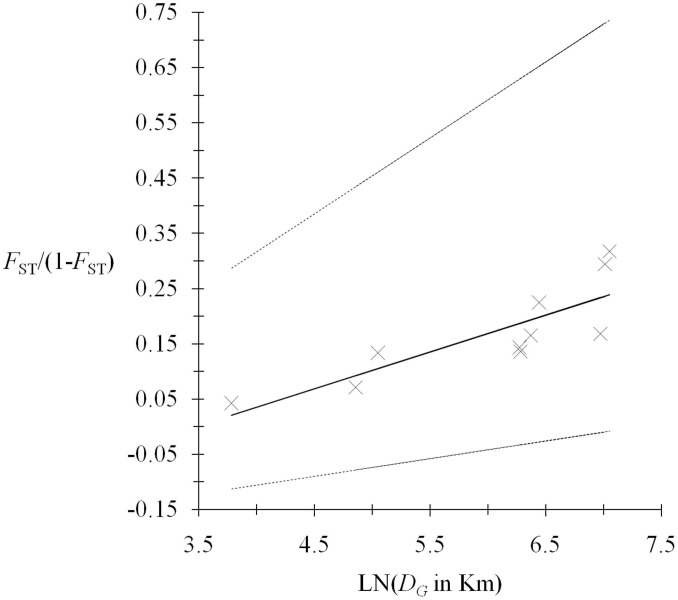
Isolation by distance between *T. evansi* from Sudan 2009. Equations are of the form *F*_ST_/(1-*F*_ST_)∼*b*LN(*D_G_*)-0.233, where *F*_ST_ is Wright's fixation index estimated with Weir and Cockerham's method and *b* is the slope of the regression and is equal to 0.067 for the main regression and *b*1  =  0.032 and *b*2  =  0.137 for the 95% confidence interval slopes. The relationship was highly significant (*P*-value  =  0.008, Mantel test, 10^6^ randomizations).
