# Supplementary material for: Population Genetics of Trypanosoma evansi from Camel in the Sudan
Source: PLoS Negl Trop Dis. 2011 Jun 7;5(6):e1196. doi: 10.1371/journal.pntd.0001196 (PMC3110163; doi:10.1371/journal.pntd.0001196)
Supplement: Supplementary Material S1 — Description of sampling designs, Wahlund protocols, dropout protocols and results of simulations for testing how Wahlund effects and/or allelic dropouts can help interpreting Trypanosoma evansi data in Sudan. (DOC) [file pntd.0001196.s001.doc]

**Supplementary material** **section**

Simulations were undertaken with Easypop V 2.01(Balloux, 2001 [61]). For the best result obtained (closest to *T. evansi* data), we simulated 100 subpopulations of 1000 purely clonal individuals each with non-overlapping generations. Migration rate was 0.001. All 15 independent loci diplayed a mutation rate of 0.0001 with a KAM mutation model with 15 possible states (alleles). Simulation began with all individuals with the same unique allele across all subpopulations and ended after 5000 generations. All 100 subpopulations were sampled with 10 individuals in each. Sampling then followed the rules described in Figure S1 and S2 for No-Wahlund and Wahlund samplings with the number of individuals corresponding to the five 2009 samples of *T. evansi*.


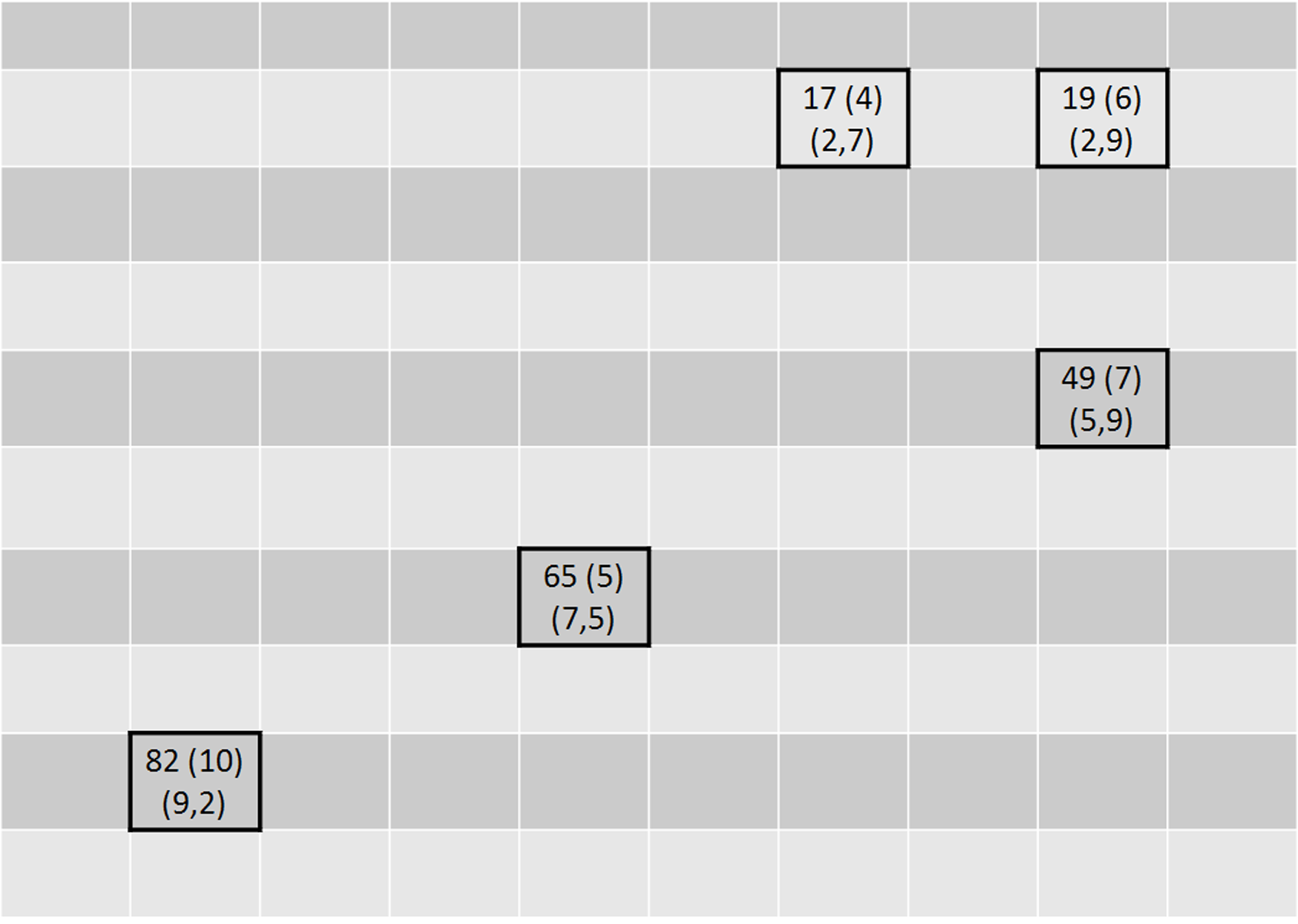


**Figure S1**: Sampling design with no Wahlund effects. Population number (after Easypop naming) is indicated first then followed, between brackets, the number of individuals sampled and the coordinates for the isolation by distance tests of Genepop 4.


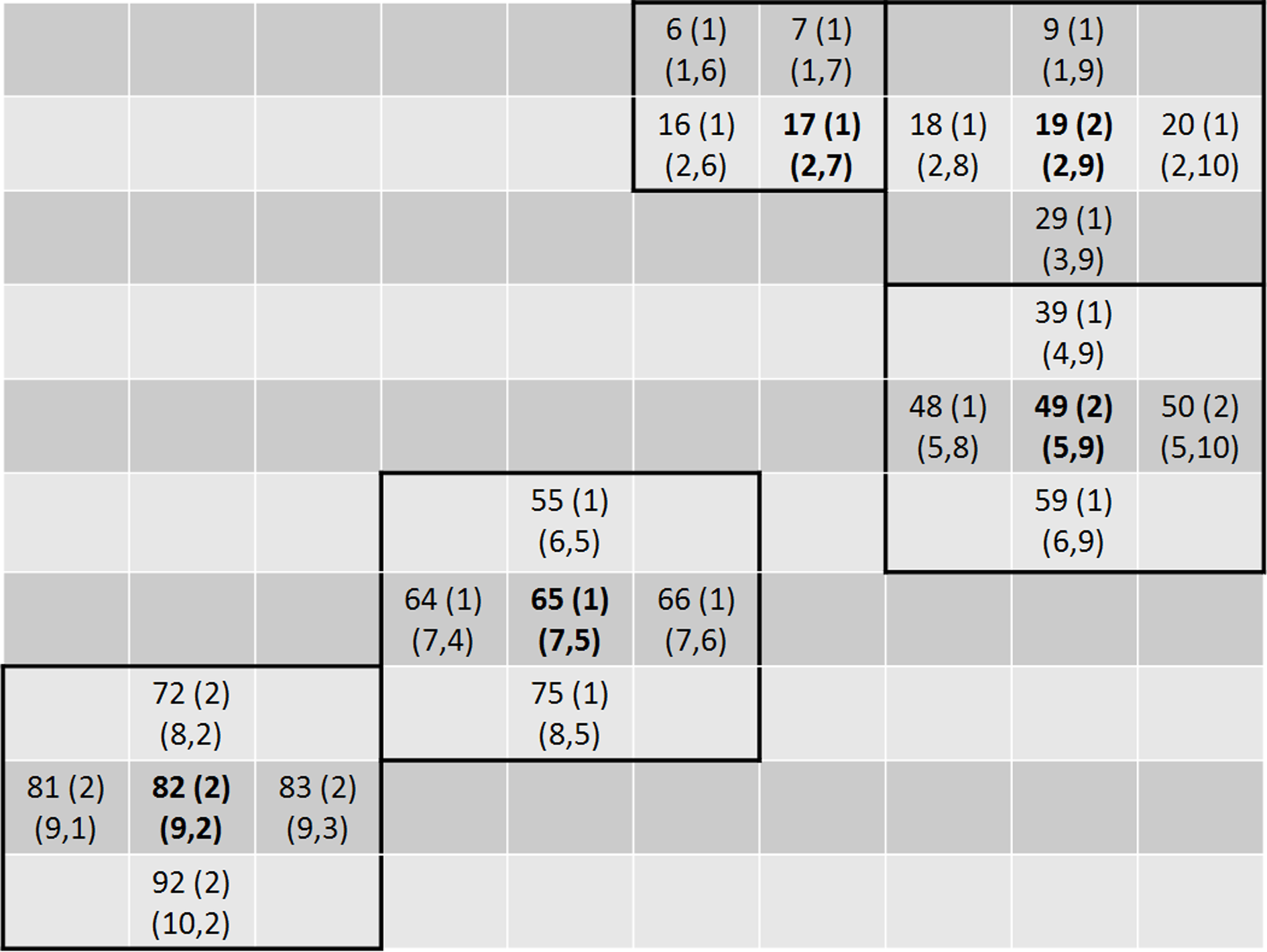


**Figure S2**: Sampling design with Wahlund effect. The numbers are the same as for Figure S1. Each Wahlund effect subsample is surrounded by a squared line. Coordinates used for the isolation by distance test are indicated in bold.

Drop out was undertaken as follows. For the no-Wahlund data, for each locus a drop out proportion was computed as *d*=(*H_ot_*-*H_o_*)/*H_o_* where *H_ot_* is the observed heterozygosity of the simulation (theoretical) and *H_o_* is the observed heterozygosity of *T. evansi* real data. For the Wahlund effect data we set
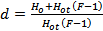
 where *F* was arbitrarily set to 0.5. For each locus in each subsample, the number of genotypes dropped out corresponded to the inferior integer corresponding to *d* times the subsample size *Ns*. Dropout was achieved by creating a homozygote for the first allele of corresponding designed genotypes. For the first locus of the first subsample we dropped out the first *dN_s_* individuals. For the same locus but the second subsamples we began with the *dN_s_*+1 individuals and so on, in order to pseudo randomize the process.

Three different analyses were then undertaken, the first with no Wahlund effect but drop out, the second with Wahlund effect but no dropout and the third with both. The results are presented in Figures S3, S4 and S5 respectively.


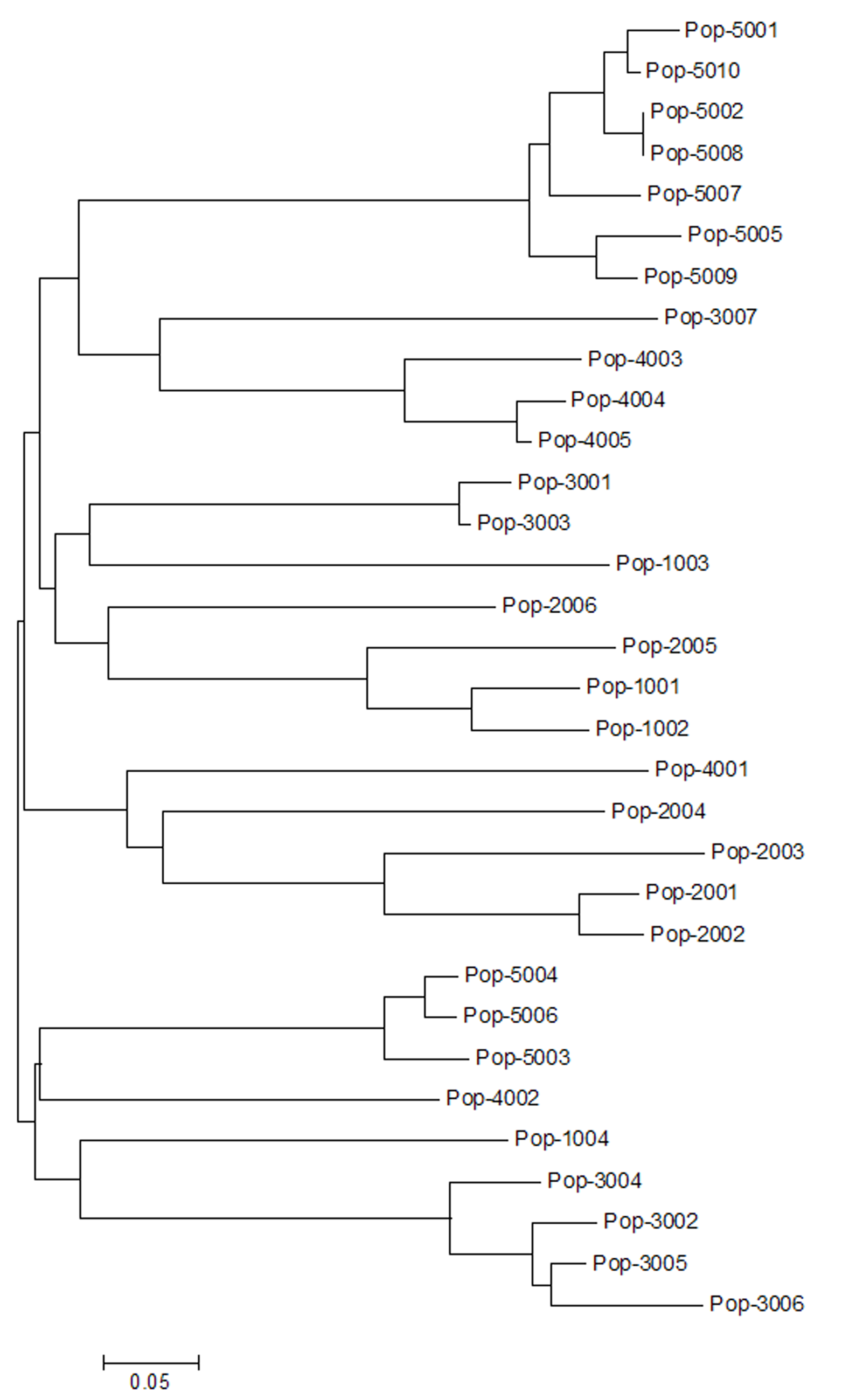


**Figure S3**: NJTree result with the no Wahlund effect but allelic dropout simulated data. First number is the population number and the second (preceded with 00) is the individual number.


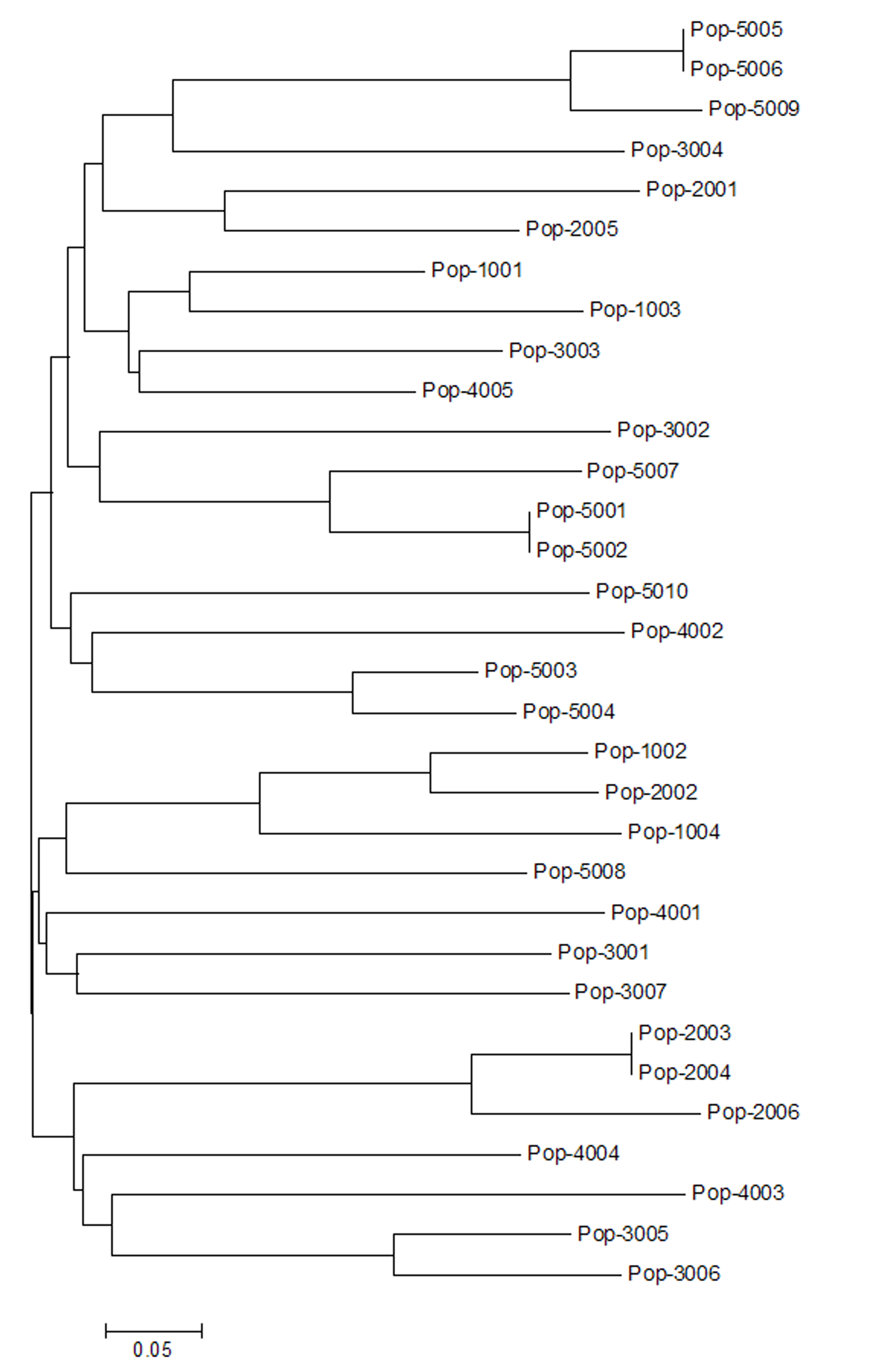


**Figure S4**: As Figure S3 for Wahlund effect data without allelic dropout.


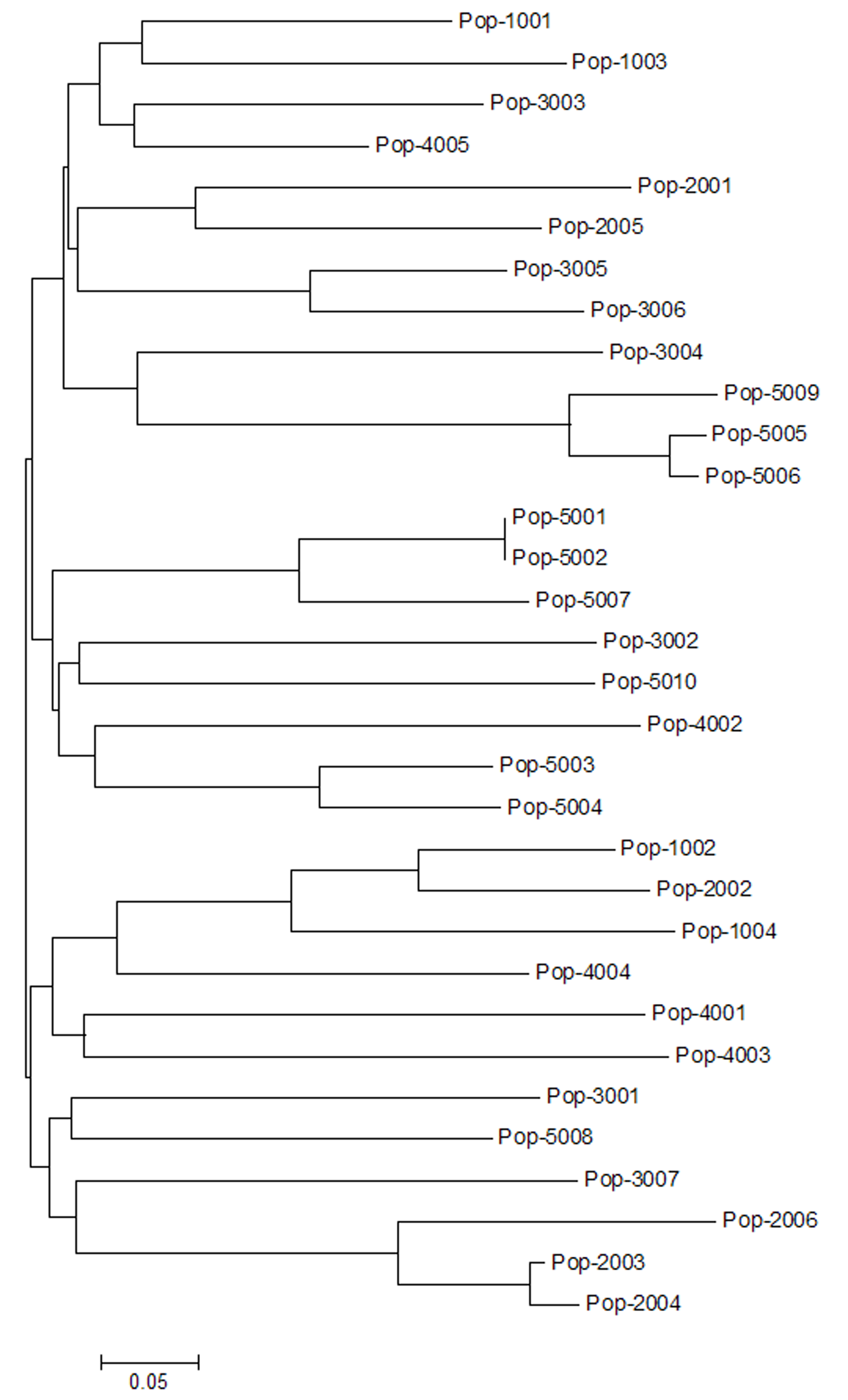


**Figure S5**: As Figure S3, for data with both Wahlund and allelic dropout.
